# New Copper-Based Metallodrugs with Anti-Invasive Capacity

**DOI:** 10.3390/biom13101489

**Published:** 2023-10-07

**Authors:** Alessia Garufi, Francesca Scarpelli, Loredana Ricciardi, Iolinda Aiello, Gabriella D’Orazi, Alessandra Crispini

**Affiliations:** 1 Department of Research and Advanced Technologies, IRCCS Regina Elena National Cancer Institute, 00144 Rome, Italy; alessia.garufi@ifo.it; 2 MAT-In_LAB, Dipartimento di Chimica e Tecnologie Chimiche, Università della Calabria, 87036 Arcavacata di Rende, CS, Italy; francesca.scarpelli@unical.it (F.S.); iolinda.aiello@unical.it (I.A.); alessandra.crispini@unical.it (A.C.); 3 CNR NANOTEC-Institute of Nanotechnology U.O.S. Cosenza, 87036 Arcavacata di Rende, CS, Italy; loredana.ricciardi@cnr.it; 4 School of Medicine, UniCamillus International University of Health Sciences, 00100 Rome, Italy; 5 Department of Neurosciences, Imaging and Clinical Sciences, University “G. D’Annunzio”, 66013 Chieti, Italy

**Keywords:** copper-based metallodrugs, tropolone, saccharine, HIPK2, cancer, invasion, wound healing

## Abstract

While metal-based complexes are deeply investigated as anticancer chemotherapeutic drugs, fewer studies are devoted to their anti-invasive activity. Herein, two copper (Cu)(II) tropolone derivatives, [Cu(Trop)Cl] and [Cu(Trop)Sac], both containing the N,N-chelated 4,4′-bishydroxymethyl-2,2′-bipyridne ligand, were evaluated for their anticancer and anti-invasive properties. RKO (RKO-ctr) colon cancer cells and their derivatives undergoing stable small interference (si) RNA for HIPK2 protein (RKO-siHIPK2) with acquisition of pro-invasive capacity were used. The results demonstrate that while [Cu(Trop)Sac] did not show cytotoxic activity, [Cu(Trop)Cl] induced cell death in both RKO-ctr and RKO-siHIPK2 cells, indicating that structural changes on substituting the coordinated chloride ligand with saccharine (Sac) could be a key factor in suppressing mechanisms of cellular death. On the other hand, both [Cu(Trop)Sac] and [Cu(Trop)Cl] complexes counteracted RKO-siHIPK2 cell migration in the wound healing assay. The synergic effect exerted by the concomitant presence of both tropolone and saccharin ligands in [Cu(Trop)Sac] was also supported by its significant inhibition of RKO-siHIPK2 cell migration compared to the free Sac ligand. These data suggest that the two Cu(II) tropolone derivatives are also interesting candidates to be further tested in in vivo models as an anti-invasive tumor strategy.

## 1. Introduction

Cancer is one of the deadliest diseases worldwide and is the second most common cause of death after cardiovascular diseases [1]. Cancer progression is the consequence of a complex multi-stage biochemical process leading to the acquisition of migratory ability by the cancer cells that, through the vascular or lymphatic circulation, reach a secondary site to form a metastasis [2,3]. For the great majority of cancer patients, a diagnosis of metastatic disease implies a terminal disease [3]. Therefore, blocking metastatic pathways holds preclinical and clinical promises for cancer patients at a risk of metastatic disease.

Multiple agents have been proven to inhibit or shrink metastases [4], and novel complexes are continuously investigated. Among those, there are metal-based complexes that, although deeply explored as anticancer chemotherapeutic drugs, are less investigated for their ability to target cancer migration and metastasis processes [5,6,7]. Within the field of the specific category of anti-invasion and antimetastatic metallodrugs (‘migrastatics’), ruthenium-based compounds have shown antimetastatic properties [4,7,8,9]. Moreover, essential metal complexes show several biological activities such as antioxidant, antimicrobial, anti-inflammatory, antiproliferative, and enzyme inhibitory. The type of organic ligands, the coordination number, and the oxidation state of the metal center in these types of complexes are highly responsible for their pharmacological activities [10,11]. In this regard, copper-based complexes have proven to be versatile compounds as candidates for antitumor, antimalarial, antituberculosis, antifungal, and anti-inflammatory drugs, particularly when based on N,N-, NO-, and O,O-chelating ligands [12,13,14]. Particular attention is paid to square-planar Cu(II) homoleptic and heteroleptic complexes, containing tropolone (Trop), N,N-diimine ligands, doxycycline, and flavonoids, as promising metallodrugs with antimetastatic activity [15,16,17].

**Figure 1:**
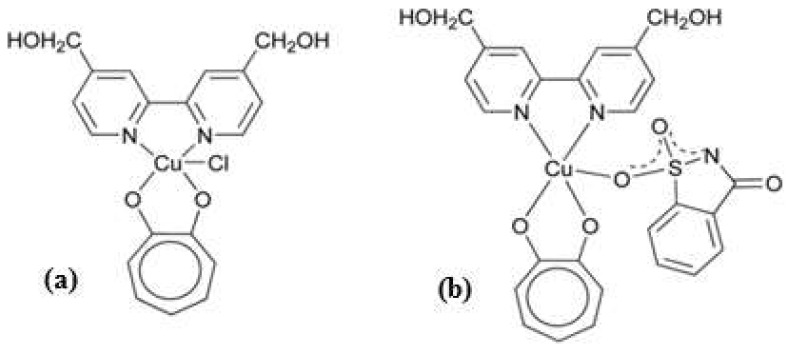
Molecular structures of the (**a**) [(bpy-OH)Cu(Trop)(Cl)]H_2_O and [Cu(Trop)Sac] (**b**) complexes.

In this study, a water-soluble heteroleptic Cu(II) derivative containing tropolonate (Trop) and saccharinate (Sac), with the general formula [(bpy-OH)Cu(Trop)Sac], (bpy-OH= 4,4′-bishydroxymethyl-2,2′-bipyridine) [Cu(Trop)Sac] [18], has been tested as a potential and virtuous molecule within the field of anti-invasion and antimetastatic metallodrugs. In this molecule, the essential metal ion, coordinated to the biologically active tropolone ligand, is found to carry a saccharine ligand in its deprotonated form through a bond with the sulfanilic oxygen atom (Figure 1). Saccharin and its derivatives are known for their ability to selectively inhibit carbonic anhydrase IX (CAIX) activity, a transmembrane homodimeric enzyme that is highly expressed in hypoxic tumor tissues and that can contribute to tumor invasion [19]. Therefore, the conjugation of this organic enzyme inhibitor to the copper center could give rise to a metal-based shuttle molecule with increased enzyme-binding selectivity and affinity when compared to the free saccharine molecule [20]. On the other hand, tropolone has shown antitumoral and anti-invasive activities [17,21,22]. In particular, the homoleptic complex Cu(Trop)_2_ has proven to exhibit both anticancer and antimetastatic properties against human breast cancer cells [17]. To evaluate the conjugated effect of tropolone and saccharine both present in the single molecule [Cu(Trop)Sac], its precursor [(bipy-OH)Cu(Trop)Cl] [Cu(Trop)Cl] [18] (Figure 1), lacking the coordinated saccharine ligand, has been investigated for comparison.

The antitumor activity of both copper complexes as well as that of the free ligand saccharine has been tested in the RKO colon cancer cell line with or without HIPK2 (homeodomain-interacting protein kinase 2) expression [23]. HIPK2 is an evolutionarily conserved protein kinase that modulates many molecular pathways involved in cell apoptosis, DNA damage response, protein stability, and protein transcription, and its deregulation contributes to cancer progression, invasion, and metastasis and reduces cancer cell response to drugs [24,25,26], other than to angiogenic and fibrotic diseases [27,28,29]. Intriguingly, the lack of HIPK2 activity induces a pseudo-hypoxic phenotype in cancer cells that contributes to cancer cell invasion [30]. As moderate concentrations of ROS can activate pro-survival pathways in cancer cells and contribute to cancer progression, while high concentrations can induce cancer cell apoptosis and contribute to tumor eradication [31], the evaluation of the antioxidant properties of the complex [Cu(Trop)Sac] has been performed in comparison to the precursor [Cu(Trop)Cl], by means of the DPPH• (2,2-diphenyl-1-picrylhydrazyl) assay method, widely used to simulate the deleterious role of free radicals in biological systems.

## 2. Materials and Methods

### 2.1. Synthesis of the Heteroleptic Copper-Based Biomolecules

The heteroleptic copper-based complexes of the general formula [(bpy-OH)Cu(Trop)Cl] H_2_O [Cu(Trop)Cl] [Cu(Trop)Cl] and [(bpy-OH)Cu(Trop)Sac] H_2_O [Cu(Trop)Sac], (bipy-OH= 4,4′-bishydroxymethyl-2,2′-bipyridine) have been synthesized as recently reported [18] (Figure 1).

### 2.2. Cell Cultures and Treatments

The human RKO colon cancer cell line (carrying endogenous HIPK2, RKO-ct) and the RKO cells stably interfered for HIPK2 function (RKO-siHIPK2) [23] were cultured in Dulbecco’s modified Eagle’s medium (DMEM) (Life Technologies-Invitrogen, Eggenstein, Germany), plus 10% heat-inactivated fetal bovine serum (FBS) (Corning Life Sciences, New York, NY, USA), glutamine, and antibiotics (Corning Life Sciences, New York, NY, USA) in a humidified atmosphere with 5% CO_2_ at 37 °C. The copper-based complexes and the ligand saccharine were dissolved in DMSO (dimethyl sulfoxide) and used at different concentrations for the indicated times.

### 2.3. Viability Assay and Cell Proliferation (XTT) Assays

Subconfluent cells were plated in triplicate in six-well Petri dishes and, the day after, treated with different concentrations of the copper complexes for 24 h. Cell viability was assessed by Trypan blue (Sigma-Aldrich, #72571, St. Louise, MO, USA) staining of both adherent and floating cells, counting blue (dead)/total cells with a Neubauer hemocytometer using light microscopy. Cell proliferation was evaluated by XTT assay using the Cell Proliferation II kit following the manufacturer’s instructions (Roche Diagnostic S.p.A., Monza, Italy), as reported [32]. Briefly, cells were seeded in 96-well culture plates (5 × 10^3^ cells/well in triplicates) and, the day after, treated with different concentrations of the complexes for the indicated time. After treatment, XTT was added for 4 h at 37 °C before stopping the formazan formation with the solubilization solution. The absorbance was measured at a wavelength of 492 nm using the Multiskan FC microplate reader (Thermo Fisher Scientific, Waltham, MA, USA).

### 2.4. Wound Healing Assay

Cells were plated in triplicates in 24-well plates at a density of 180,000 (RKO-ctr) or 250,000 (RKO-siHIPK2) cells per well, forming a single cell layer the day after. At 24 h after plating, cells were scratched through the monolayer using a 1000 µL pipette tip. Cells were rinsed to remove cellular debris, and serum-free medium was added before placing plates in an IncuCyte Live Analysis Incubator (Essen BioScience, Ann Arbor, MI, USA) at 37 °C. The IncuCyte live-cell imaging enables noninvasive, full kinetic measurements of cell growth based on area (confluence) metrics. Images of the wells were taken every 6 h, and migration assays were analyzed until 66 later. For drug treatment, media containing the specific concentration of the copper complexes were added at the time of the scratch. The ligand saccharine (Sac) was used as control. Data were analyzed by using the IncuCyte software v2022A package (Essen BioScience, Ann Arbor, MI, USA). The percentage (%) of migration was calculated using the following formula: 100 − (final area/initial area × 100%).

### 2.5. Western Blot Analysis

Cells were harvested, centrifuged, and the resulting pellets lysed in lysis buffer (150 mM NaCl, 50 mM Tris–HCl, pH 7.5, 150 mM KCl, 1 mM dithiothreitol, 5 mM EDTA, and 1% Nonidet P-40) (all from Sigma-Aldrich, St. Louise, MO, USA) plus protease inhibitors (CompleteTM, Mini Protease Inhibitor Cocktail, Merck, Life Science S.r.l., Milan, Italy). Protein concentration was determined by using a BCA (bicinchoninic acid assay) protein assay (Sigma-Aldrich, St. Louise, MO, USA, cat. n.71285-M). Equal amount of total cell lysates was separated on denaturing 8–15% SDS-PAGE (polyacrylamide gel electrophoresis) gels (Bio-Rad, Hercules, CA, USA). After electrophoresis, proteins underwent semidry blotting to polyvinylidene difluoride (PVDF) membranes (Immobilon-P, Merk-Millipore, Milan, Italy). Membranes were blocked in Tris-buffered saline containing 0.1% Tween 20 (TBS-T) and 3% BSA (Merck-Sigma-Aldrich, Darmstadt, Hesse, Germany) for 30 min at room temperature (RT), incubated with the primary antibodies overnight at 4 °C and then probed with the appropriate secondary antibodies coupled to horseradish peroxidase (HRP) (Bio-Rad Laboratories, Segrate, Italy). Enzymatic signal was visualized by chemiluminescence (ECL Detection system, Amersham GE Healthcare, Milan, Italy). The following antibodies were used: rabbit polyclonal anti-phospho-4E-BP1 (Thr37/46) (1:200) (cat. n. 2855) and rabbit polyclonal anti-4E-BP1 (1:200) (Cell Signaling, Danvers, MA, USA, cat. n. 9452). Mouse monoclonal β-actin (Sigma-Aldrich, St. Louise, MO, USA, cat. n. A5441) was used as protein loading control. Signals evidenced by Western blot were subjected to densitometry by using ImageJ software, which was downloaded from the NIH website (available online at https://imagej.nih.gov, accessed on 10 February 2022), and relative band intensity normalized to β-actin and plotted as protein expression/β-actin ratio.

### 2.6. Measurement of Intracellular Reactive Oxygen Species (ROS) Production

2,2-Diphenyl-1-picrylhydrazyl radical (DPPH) actin (Sigma-Aldrich, St. Louise, MO, USA, cat. n. D9132) was dissolved in spectrophotometric grade ethanol to prepare a 1.2 × 10^−4^ M solution. Then, 5 mL of the obtained solution was added to 5 mL of [Cu(Trop)Cl] and [Cu(Trop)Sac] ethanolic solution (3.2 × 10^−4^ M) and left at room temperature in the dark. Absorption spectra were recorded after 3, 24, 48, and 72 h using a Perkin Elmer Lambda 900 spectrophotometer. The antioxidant activity of [Cu(Trop)Cl] and [Cu(Trop)Sac] compounds was evaluated by monitoring the decrease of DPPH optical density at 517 nm according to the following equation:

$$\%\;Antioxidant\;activity=\frac{A_{0}{-A}_{s}}{A_{0}}\cdot 100$$

where *A*_0_ is the absorbance of the control (DPPH ethanolic solution), and *A_S_* is the absorbance of the working solution (DPPH ethanolic solution mixed with [Cu(Trop)Cl] and [Cu(Trop)Sac] solutions).

### 2.7. Stability Measurements

[Cu(Trop)Cl] and [Cu(Trop)Sac] were dissolved in DMSO and then diluted to reach a final concentration of 1 × 10^−5^ M in DMSO/buffer 0.5% *v*/*v*. The buffer solution (pH 7.4) was prepared by dissolving one phosphate-buffered saline table in 200 mL of water.

### 2.8. Statistical Analysis

The results are expressed as mean ± standard deviation (S.D.) of at least three independent experiments, and a two-tailed Student’s *t*-test was applied to demonstrate statistical significance. Differences were considered statistically significant when the *p*-value was at least ≤0.05.

## 3. Results and Discussion

### 3.1. Stability of [Cu(Trop)Cl] and [Cu(Trop)Sac] Complexes in Solution

**Figure 2:**
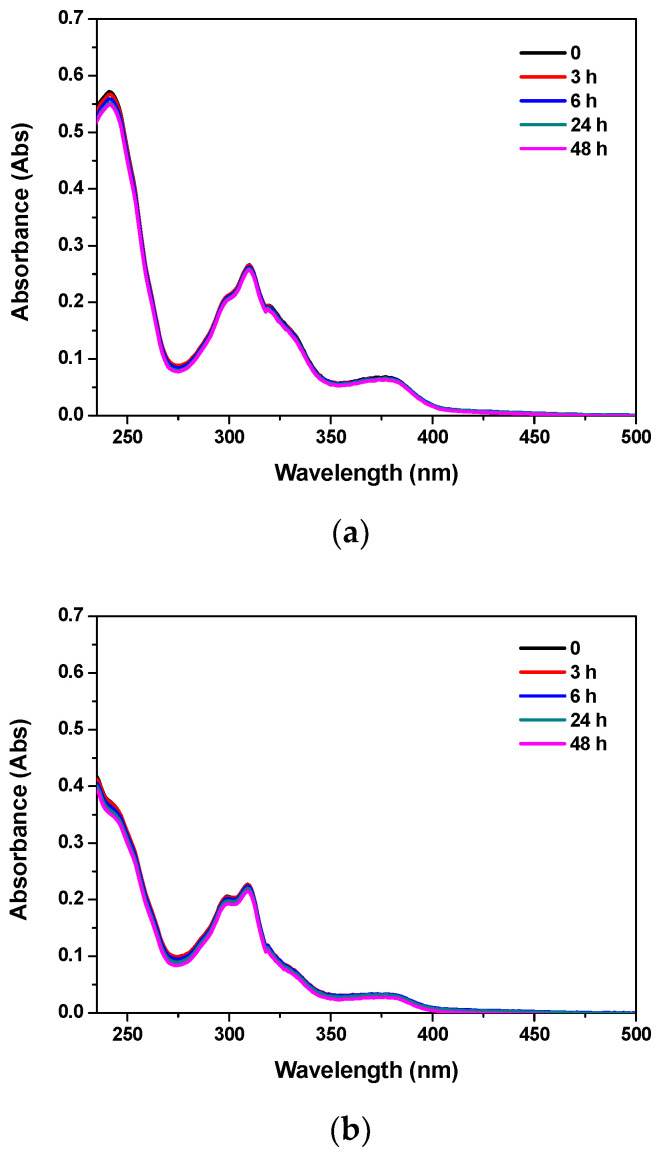
Absorption spectra over time (*t* = 0, 3, 6, 24 and 48 h) of [Cu(Trop)Cl] (**a**) and [Cu(Trop)Sac] (**b**) complexes in DMSO/buffer solution (DMSO 0.5% *v*/*v*) at room temperature, 1 × 10^−5^ M.

To assess spectroscopically the long-term stability of [Cu(Trop)Cl] and [Cu(Trop)Sac] complexes in aqueous solution, electronic spectra were recorded at times ranging from 0 to 48 h at room temperature (Figure 2).

As clearly shown in Figure 2, no significant changes were observed in the absorption profiles within the time-lapse, indicating that both compounds were stable under experimental conditions.

### 3.2. Inhibition of RKO-siHIPK2 Cell Proliferation by [Cu(Trop)Sac] Treatment

**Figure 3:**
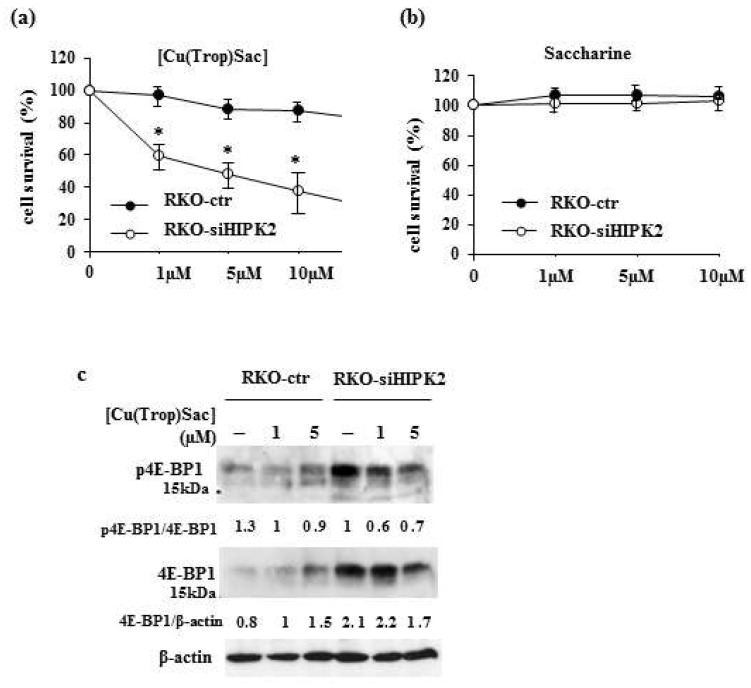
Anticancer activity of the [Cu(Trop)Sac] complex. RKO-ctr and RKO-siHIPK2 colon cancer cells were left untreated or treated with the indicated amount of (**a**) [Cu(Trop)Sac] complex or (**b**) saccharine for 24 h before measuring cell survival by XTT assay. (**c**) Western blot analysis of the indicated proteins in RKO-ctr and RKO-siHIPK2 colon cancer cells left untreated or treated with the indicated amount of [Cu(Trop)Sac] complex for 24 h (original images can be found in Figure S1). The ratio of protein levels vs. βactin, used as a protein loading control, was measured by densitometric analysis and is reported. * *p* ≤ 0.05.

The anticancer effect of [Cu(Trop)Sac] was evaluated in RKO colon cancer cells undergoing control small interference (si) RNA (RKO-ctr) and in its derivative undergoing stable small interference (si) RNA for HIPK2 protein (RKO-siHIPK2) [23]. Cell proliferation and cell viability were first evaluated in RKO-ctr and RKO-siHIPK2 cancer cells treated with different doses of [Cu(Trop)Sac] for 24 h via XTT and trypan blue assays. The results of the XTT assay showed that [Cu(Trop)Sac] treatment significantly impaired RKO-siHIPK2 cell proliferation in a dose-dependent way while only slightly impairing the cell proliferation of RKO-ctr cells (Figure 3a). These data suggest a greater sensitivity of the HIPK2-interfered cells to the antiproliferative effect of the complex. The treatment with the ligand saccharine (Sac) did not modify the proliferation of both cell lines (Figure 3b), hence revealing the important role of its presence as a coordinated ligand within the [Cu(Trop)Sac] complex. Viability by Trypan blue staining did not show any cell death of both RKO-siHIPK2 and RKO-ctr cells in response to [Cu(Trop)Sac] treatment, indicating lack of cytotoxic activity of the complex at the used concentrations and in this setting. At the molecular level, the phosphorylation of the 4E-BP1 molecule, the target of the mTOR pro-survival pathway, was evaluated since its activation can sustain cancer cell survival and predict poor prognosis [33]. In addition, hyperphosphorylation of 4E-BP1 is associated with malignant progression and poor prognosis [33,34]. The results show that the HIPK2-interfered cells presented, at the basal level, a greater level of 4E-BP1 protein compared to the control cells (Figure 3c). The treatment with [Cu(Trop)Sac] decreased the phosphorylation of 4E-BP1 to a greater extent in the HIPK2-interfered cells compared to the control cells (Figure 3c).

### 3.3. Inhibition of RKO-HIPK2i Migration by [Cu(Trop)Sac] Treatment

**Figure 4:**
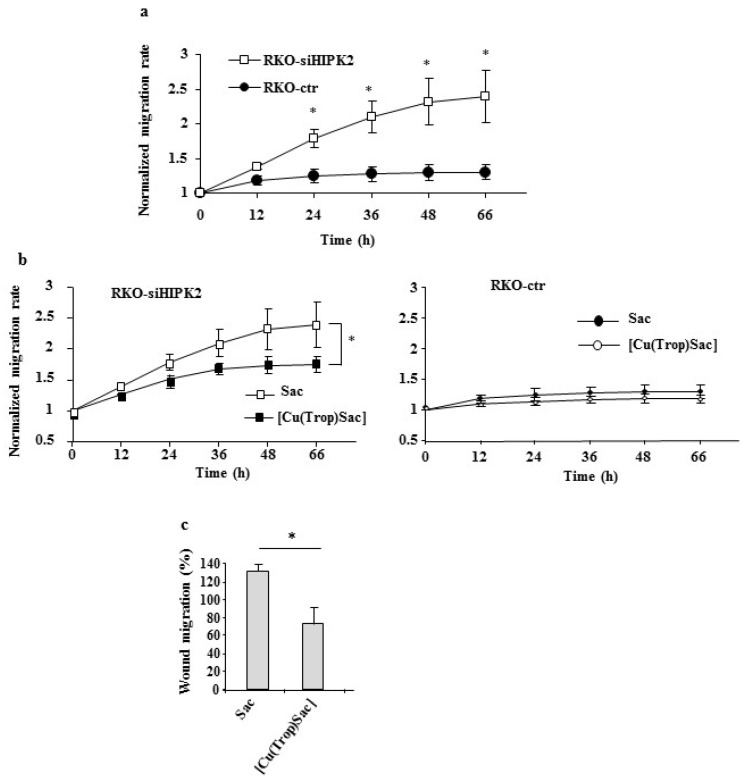
Cell migration following [Cu(Trop)Sac] treatment. (**a**) Wound healing assay on RKO-ctr and RKO-siHIPK2 during time after scratch. Wound healing assay following [Cu(Trop)Sac] or saccharine (Sac) (5 µM) treatment on ((**b**), **left panel**) RKO-siHIPK2 and ((**b**), **right panel**) RKO-ctr cells. (**c**) Histograms showing the percentage of RKO-siHIPK2 cell migration treated as in (**b**). * *p* ≤ 0.05.

Dephosphorylation of 4E-BP1 has been shown to be an important biomarker for predicting cancer cell response to anticancer therapies [35] and for reducing cell invasion by, for instance, inhibiting the epithelial–mesenchymal transition (EMT) [36]. Therefore, the effect of [Cu(Trop)Sac] on cell migration was next evaluated by wound healing assay. The results show that the RKO-siHIPK2 cells had, at the basal level, a faster migration capacity compared to the control cells (Figure 4a); treatment with the [Cu(Trop)Sac] significantly inhibited the cell migration capacity of RKO-siHIPK2 cells compared to the ligand saccharine (Sac) (Figure 4b, left panel), while did not modify the migration ability of the RKO-ct cell line (Figure 4b, right panel). At 5 μM, the complex [Cu(Trop)Sac] reduced the RKO-siHIPK2 wound area by about 56% (Figure 4c) over saccharine conditions.

These results are in line with the antimetastatic activity of an analogous copper(II)-tropolone complex against breast cancer cells [17]. Moreover, since tropolone and uncoordinated bipyridine ligands, as well as Cu(II) metal cation, require very high concentrations to show any anticancer and antiproliferative activities [17,37,38,39], combined with the fact that no dissociation of ligands is observed in the experimental studied conditions, complexation represents the key role in the antimetastatic activity of [Cu(Trop)Sac] complex.

The results also underline the key role of HIPK2 in regulating molecular mechanisms able to restrain cancer cell migration ability. Thus, inhibition of HIPK2 activity has been associated with increased metastatic potential of different cancer types by, for instance, inhibiting the EMT [40,41,42,43,44]. In addition, HIPK2 regulates the hypoxia-inducible factor-1 (HIF-1) activity that promotes cancer cell angiogenesis and invasion [28,29,30]. Hypoxia is a condition commonly seen in metastatic tumors where cells are deprived of oxygen due to rapid proliferation and a shift in their metabolism [45]. Among the HIF-1-induced target genes is carbonic anhydrase IX (CAIX), whose overexpression has been associated with increased tumor progression [19,46], becoming a potential drug target to inhibit cancer invasion and metastasis [47]. Of note, saccharin and its derivatives have been shown to selectively target carbonic anhydrase IX (CAIX) activity, being considered a promising class of metalloenzyme inhibitors [19]. Therefore, it is tempting to speculate that [Cu(Trop)Sac] may impair the metastatic potential of cancer cells by targeting the hypoxia-inducible pathways, although this hypothesis needs to be further supported. However, since, among others, enzyme inhibition is a proposed mechanism for the anticancer and antiproliferative activities of several metal complexes, including copper-containing derivatives [48,49], it cannot be excluded that the results obtained for [Cu(Trop)Sac] can derive from synergic effects due to the concomitant presence of the Cu(II) ion and both coordinated saccharine and tropolone ligands.

### 3.4. Anticancer and Antiproliferative Activities of [Cu(Trop)Cl]

**Figure 5:**
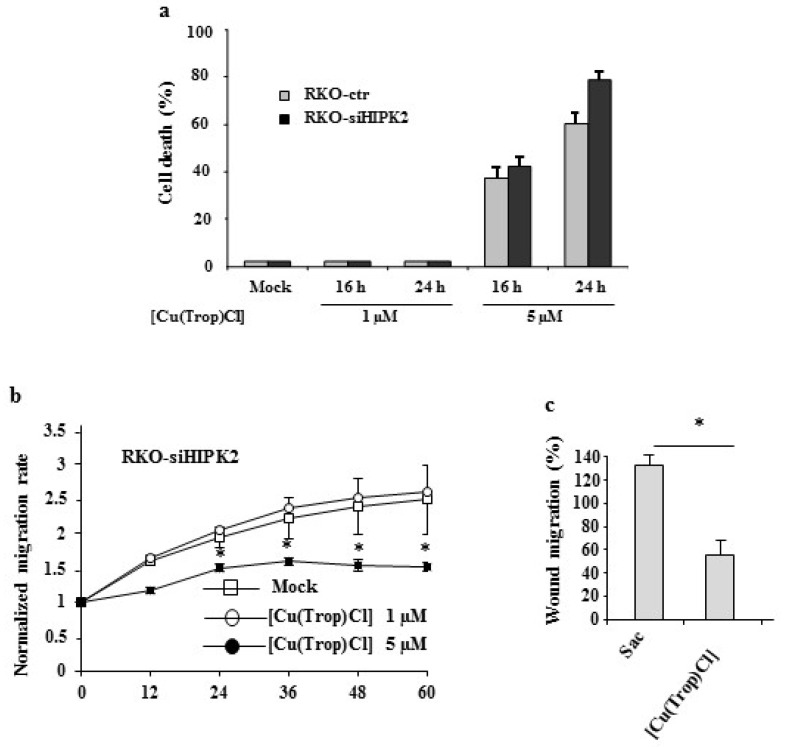
Anticancer activity of the [Cu(Trop)Cl] precursor. RKO-ctr and RKO-siHIPK2 colon cancer cells were left untreated or treated with the indicated amount of (**a**) [Cu(Trop)Cl] precursor for 16 and 24 h before measuring cell viability by Trypan blue assay. (**b**) Wound healing assay on RKO-siHIPK2 cells mock-treated or treated with [Cu(Trop)Cl] (1 and 5 µM) for the indicated time. (**c**) Histograms showing the percentage of RKO-siHIPK2 cell migration treated as in (**b**). * *p* ≤ 0.05.

To evaluate the single role of the saccharin ligand in the anticancer and antiproliferative activities of this class of Cu(II) complexes, the precursor [Cu(Trop)Cl], lacking the coordinated saccharine ligand, has been investigated for comparison. Treatment with [Cu(Trop)Cl] induced cell death in both RKO-ctr and RKO-siHIPK2 cells (Figure 5a) in a time-dependent manner, proving that, differently than [Cu(Trop)Sac], its precursor showed cytotoxic activity at the used concentrations These results are in line with the recently reported cytotoxic activity against several cancer cell lines of the analogous Cu(II) tropolone derivative of formula [Cu(2,2′-bpy)TropCl]H_2_O, whose mechanism of action has been proposed to be the binding to double-stranded DNA through intercalation [39]. These findings suggest that the substitution of the chloride ligand with the saccharinate is not innocent in the cytotoxic activity of these species, most probably related to a mechanism of action suppressed in the case of [Cu(Trop)Sac]. Indeed, since [Cu(Trop)Cl] is found as an ionic species in solution, due to the labile coordination of the chloride ligand to the Cu(II) ion, the overall planar structure of the remaining [CuTrop]^+^ cation could favor cytotoxic activity through intercalation mode. On the contrary, the structural characterization of [Cu(Trop)Sac] has proven the strong coordination of the saccharine ligand to the Cu(II) center, found a neutral species in solution, with an overall slightly distorted square-pyramidal geometry, where the two chelated ligands, bipy-OH and Trop, occupy the basal plane. Moreover, the computational analysis performed on [Cu(Trop)Sac] has revealed that the Sac ligand can only be substituted in the presence of competing ligands, such as N- and S-donor residues of proteins and peptides, releasing saccharine in the physiological environment [18]. Therefore, it is reasonable to propose that the coordination of saccharin to the Cu(II) ion impedes the [CuTrop]^+^ residue from forming and acting as an intercalating agent. On the contrary, the analysis of RKO-siHIPK2 cell migration ability demonstrated that [Cu(Trop)Cl] counteracted migration in the wound healing assay (Figure 5b), and, in particular, at 5 μM, [Cu(Trop)Cl] reduced the RKO-siHIPK2 wound area of about 58% (Figure 5c) over mock conditions.

The antiproliferative activity, already exerted by the complex precursor, is then preserved in [Cu(Trop)Sac], proving that its behavior is due to the combination of both tropolone- and saccharin-coordinated ligands.

### 3.5. Antioxidant Activity of [Cu(Trop)Sac] and [Cu(Trop)Cl Complexes

**Figure 6:**
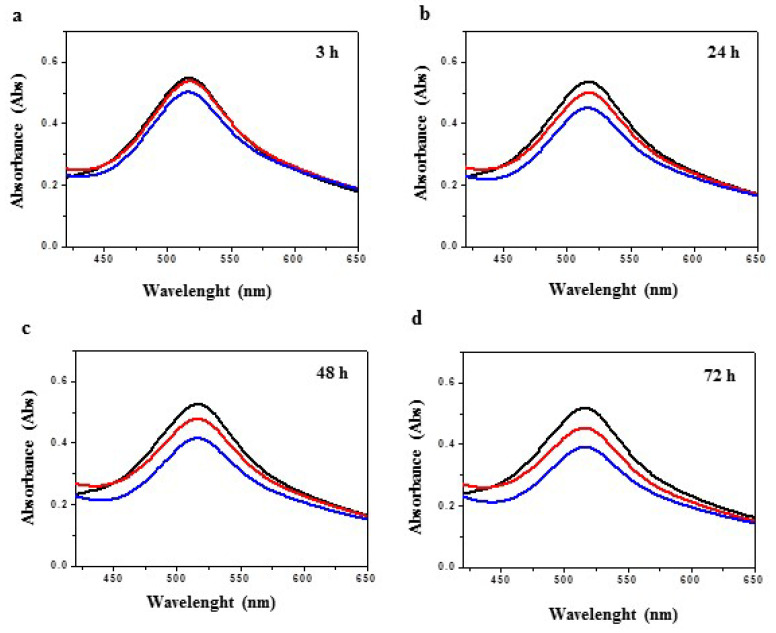
Absorption spectra of DPPH (control, black line), [Cu(Trop)Cl] (red line), and [Cu(Trop)Sac] (blue line) ethanolic solution after incubation for 3 (**a**), 24 (**b**), 48 (**c**), and 72 (**d**) hours.

A DPPH scavenging method [50,51] was used to assess the antioxidant activity of both [Cu(Trop)Cl] and [Cu(Trop)Sac] compounds. The absorption spectra of DPPH, [Cu(Trop)Cl] and [Cu(Trop)Sac], in an ethanol solution, were recorded after 3, 24, 48, and 72 h (Figure 6, Table 1) to evaluate a potential time-dependence of the DPPH radical scavenging activity. As shown in Figure 5a, after 3 h of incubation, both compounds caused a decrease of the absorption band at 517 nm, resulting in DPPH scavenging activity values of 1.84 ± 0.15 and 8.29 ± 0.13, respectively (Table 1). Extending the incubation period to 24, 48, and 72 h, an enhancement of the antioxidant activity was observed for both complexes, reaching values of 6.75 ± 0.23, 9.06 ± 0.12 and 12.62 ± 0.21 in the case of the precursor complex [Cu(Trop)Cl] and 15.78 ± 0.16, 21.02 ± 0.20 and 24.48 ± 0.18 for complex [Cu(Trop)Sac] (Figure 5b,c, Table 1). In both cases, the DPPH radical scavenging activity has been found to be time-dependent, with the maximum values reached after 72 h. However, some significant differences are found between the two species, most probably in relation to the substitution of the chloride ligand with the saccharinate one.

The antioxidant activity of complex [Cu(Trop)Sac] is found to be more relevant with respect to that of its precursor, showing a 4.5 times higher value than that of complex [Cu(Trop)Cl] after 3 h of incubation. Oxidative stress can contribute to both cancer progression and cancer cell apoptosis, according to the cancer cell concentrations of ROS [31], and targeting oxidative stress is an important anticancer strategy.

## 4. Conclusions

Within the field of the design of copper-based drugs with potential therapeutical applications, the evaluation of the antitumor and antiproliferative activities of two Cu(II) tropolone derivatives, [Cu(Trop)Sac] and [Cu(Trop)Cl], both containing the N,N-chelated 4,4′-bishydroxymethyl-2,2′-bipyridine ligand, has been performed using the RKO (RKO-ctr) colon cancer cells and its derivative undergoing stable small interference (si) RNA for HIPK2 protein (RKO-siHIPK2), the latter one with acquisition of pro-invasive capacity. The results show that while [Cu(Trop)Sac] lacks cytotoxic activity, with no significant cell death of both RKO-siHIPK2 and RKO-ctr cells, treatment with [Cu(Trop)Cl] induced cell death in both RKO-ctr and RKO-siHIPK2 cell lines, indicating that the structural changes on substituting the coordinated chloride ligand with the saccharine one could be a key factor in suppressing and/or promoting mechanisms of cellular death. On the other hand, the analysis of RKO-siHIPK2 cell migration ability demonstrated that both [Cu(Trop)Sac] and [Cu(Trop)Cl] counteracted migration in the wound healing assay. The synergic effect exerted by the concomitant presence of both coordinated tropolone and saccharin ligands in [Cu(Trop)Sac] is also supported by its significant inhibition capacity of RKO-siHIPK2 cell migration compared to the free Sac ligand. Moreover, both [Cu(Trop)Sac] and [Cu(Trop)Cl] show time-dependent antioxidative activity, with the maximum values reached after 72 h, even if [Cu(Trop)Sac] is found to be 4.5 times more active than [Cu(Trop)Cl] already after 3 h of incubation. Additional studies are needed to unveil the molecular mechanisms triggered by the complexes for their anticancer activity, such as enzyme inhibition or the targeting of the hypoxic phenotype, that are associated with cancer invasion and metastasis. In summary, the results of this study indicate that the two Cu(II) tropolone derivatives are interesting candidates to be tested in in vivo models as an anti-invasive tumor strategy.

## Figures and Tables

**Table 1:** DPPH scavenging ability of [Cu(Trop)Cl] and [Cu(Trop)Sac].

Compound	Antioxidant Activity (%) 3 h	Antioxidant Activity (%) 24 h	Antioxidant Activity (%) 48 h	Antioxidant Activity (%) 72 h
[Cu(Trop)Cl]	1.84 ± 0.15	6.75 ± 0.23	9.06 ± 0.12	12.62 ± 0.21
[Cu(Trop)Sac]	8.29 ± 0.13	15.78 ± 0.16	21.02 ± 0.20	24.48 ± 0.18

## Data Availability

The datasets generated and analyzed during the current study are available from the corresponding author upon reasonable request.

## Supplementary Materials

The following supporting information can be downloaded at: https://www.mdpi.com/article/10.3390/biom13101489/s1, Figure S1: original images.
