# Supplementary figures and images for: New Copper-Based Metallodrugs with Anti-Invasive Capacity
Source: Biomolecules. 2023 Oct 7;13(10):1489. doi: 10.3390/biom13101489 (PMC10604694; doi:10.3390/biom13101489)

**Figure S1**

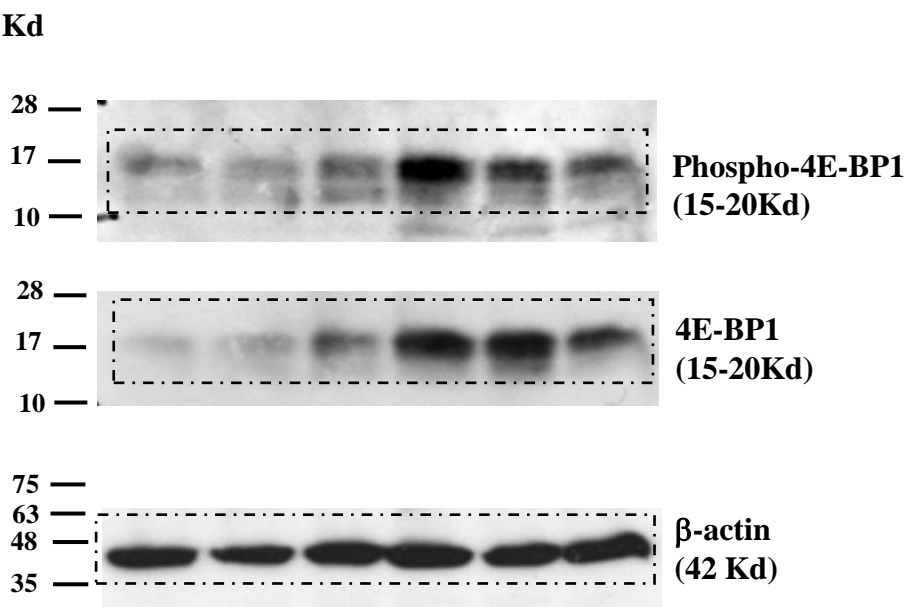

Supplement: Supplementary file 1 [file biomolecules-13-01489-s001.zip › biomolecules-2619238- Supplementary material.pdf]
